# Analytical Solutions for Electroosmotic Flow and Heat Transfer Characteristics of Nanofluids in Circular Cylindrical Microchannels with Slip-Dependent Zeta Potential Considering Thermal Radiative Effects

**DOI:** 10.3390/mi16010063

**Published:** 2025-01-05

**Authors:** Zouqing Tan, Xiangcheng Ren

**Affiliations:** School of Mechanical Engineering and Rail Transit, Changzhou University, Changzhou 213164, China; 13306116581@163.com

**Keywords:** cylindrical microchannels, slip-dependent zeta potential, radiative heat transfer, nanoparticle volume fraction, Nusselt number

## Abstract

This study analyzes the impact of slip-dependent zeta potential on the heat transfer characteristics of nanofluids in cylindrical microchannels with consideration of thermal radiation effects. An analytical model is developed, accounting for the coupling between surface potential and interfacial slip. The linearized Poisson–Boltzmann equation, along with the momentum and energy conservation equations, is solved analytically to obtain the electrical potential field, velocity field, temperature distribution, and Nusselt number for both slip-dependent (SD) and slip-independent (SI) zeta potentials. Subsequently, the effects of key parameters, including electric double-layer (EDL) thickness, slip length, nanoparticle volume fraction, thermal radiation parameters, and Brinkman number, on the velocity field, temperature field, and Nusselt number are discussed. The results show that the velocity is consistently higher for the SD zeta potential compared to the SI zeta potential. Meanwhile, the temperature for the SD case is higher than that for the SI case at lower Brinkman numbers, particularly for a thinner EDL. However, an inverse trend is observed at higher Brinkman numbers. Similar trends are observed for the Nusselt number under both SD and SI zeta potential conditions at different Brinkman numbers. Furthermore, for a thinner EDL, the differences in flow velocity, temperature, and Nusselt number between the SD and SI conditions are more pronounced.

## 1. Introduction

Micro/nanochannels have acquired notable recognition owing to their wide-ranging applications in microelectromechanical systems (MEMS), medical and clinical diagnostics, and thermal management of electronic devices [1,2,3,4]. The defining dimensions of these channels typically range from the nanometer to micrometer scale, which enables a significant enhancement in fluid transport efficiency and precise control. Fluid flow is generally driven by factors such as pressure, surface tension, external electric fields, and magnetic fields [5,6]. Among these mechanisms, the combination of pressure and external electric fields is widely utilized in micro/nanochannels [7,8]. Additionally, the use of microfluidic devices to analyze heat transfer characteristics has attracted substantial attention in the field of thermal engineering [9]. Therefore, understanding the transport dynamics in micro/nanochannels is critical for optimizing the performance of various applications.

Several researchers have found that slip boundary conditions must be introduced when fluid flows along the liquid–solid interface [10,11,12]. Shojaeian and Kosar [13] investigated the heat transfer characteristics and other related flow features in fluid flow between parallel plates under the velocity slip boundary condition. Their findings indicated that a higher slip coefficient improves the Bejan number. Kundu and Saha [14] analyzed the velocity distributions and thermal behavior of fluids under several different boundary conditions, assuming uniform heat flux at the wall. Moreover, interfacial slip on hydrophobic surfaces affects the zeta potential. When the wall of a microchannel is composed of hydrophobic material, flowing fluid causes water molecules to preferentially organize into ordered structural layers due to hydrophobic interactions. This orderly arrangement diminishes the interactions between water molecules and the wall, thereby enhancing the slip effect [15]. The amplification of the slip effect leads to an increased fluid velocity in the vicinity of the wall. The elevated flow velocity modifies the charge distribution at the interface, as fluid motion influences the rearrangement and accumulation of charges. Ultimately, these alterations in charge distribution directly affect the zeta potential [16]. Churaev et al. [17] introduced a straightforward correlation between interfacial slip length and zeta potential in KCl solution. For symmetric electrolyte solutions, Tandon et al. [16] later proposed a nonlinear relationship connecting slip length and zeta potential. Based on this nonlinear relationship, Soong et al. [18] theoretically studied electroosmotic flow considering the SD zeta potential. Banerjee et al. [19] investigated the thermal characteristics in two-dimensional planar microchannels by using a combined driving mechanism of mixed electric field and pressure and found that the SD surface potential significantly affects the heat transfer and identified the conditions under which the transition point of the Nusselt number occurs between the SD and SI conditions. Subsequently, considering the SD zeta potential, Siva et al. [20] investigated the fluid magnetohydrodynamic transport in microchannels under combined electrical and magnetic fields. Further, they extended their research [21] to explore how the SD zeta potential influences the flow behavior and transport properties of couple stress fluids in rotating microchannels. This study concluded that the SD zeta potential significantly affects the spin velocity and volumetric flow rate, with both being influenced by parameters like slip and rotational factors. All of the aforementioned studies demonstrated that the SD zeta potential significantly influenced the fluid and heat transport characteristics.

In industrial applications, nanofluids exhibit significantly better thermal properties than traditional fluids, which has attracted considerable research interest [22,23,24,25]. Nanoparticles in nanofluids typically range from 1 to 100 nanometers in scale. The thermal conductivity of materials such as Cu exceeds that of water by a factor of 700. Choi and Eastman [26] first employed the nanofluid model to examine the thermal conductivity of Cu-based nanofluids. They later extended their work [27] by improving the Maxwell equations to accurately account for the influence of examining the nanolayer on the effective thermal conductivity of solid/liquid hybrid materials. Xie and Jian [28] further studied the issues related to the energy conversion of the volume fraction of magnetic nanoparticles in microchannels and found a more appropriate volume fraction interval to enhance this effect. Later, Deng [29] added a power-law nanofluid into a rectangular channel based on heat transfer studies and found that higher nanoparticle volume fractions notably decreased velocity while increasing temperature. Afterward, Javaria et al. [30] examined the thermal transfer properties of a silver nanofluid suspended in water flowing through an asymmetric channel under electroosmosis and peristalsis. Abbasi et al. [31] conducted a thermodynamic analysis of electroosmotic flow in an Fe_3_O_4_-Cu/H_2_O hybrid nanofluid, revealing that the addition of nanoparticles reduced both temperature and entropy generation in the hybrid nanofluid. In addition, dynamic light scattering (DLS) is a widely used technique to study the nanofluids [32]. Unlike zeta potential measurements, DLS provides information not only on the hydrodynamic diameter of particles but also on the aggregation behaviors under various conditions. Stetefeld et al. [33] discussed the diverse applications of DLS in investigating the homogeneity of macromolecular solutions, molecular interactions, and supporting other analytical methodologies. Koutras et al. [34] used DLS technology to study the online stability of TiO_2_ nanoparticles in natural ester oil, identifying the optimal concentration range for these nanoparticles. Based on these findings, they further extended their research [35] by being the first to systematically assess the impact of titanium dioxide and silicon carbide nanoparticles on the stability and dielectric properties of natural ester oil nanofluids under high-temperature conditions, employing DLS and electrical testing techniques. The aforementioned studies demonstrated that nanofluids significantly impact heat transfer in fluid flow within micro/nanochannels.

In addition to nanofluids, another important factor is thermal radiation [36]. Thermal radiation, as a significant mode of energy transfer between solids and fluids, can notably influence the energy exchange process, particularly under high-temperature conditions. Nanofluids, with their unique thermal properties, such as enhanced thermal conductivity and complex microstructures, exhibit more pronounced radiative effects compared to conventional fluids [37]. In microchannels, due to the constraints of scale and flow characteristics, thermal radiation may compete with or interact with other heat transfer mechanisms, such as conduction and convection. This effect becomes especially relevant in scenarios with small temperature gradients and low Reynolds numbers, where thermal radiation can become a crucial heat transfer mode [38]. Therefore, understanding the role of thermal radiation in these complex flow and heat transfer processes is essential for a comprehensive understanding of microscale heat transfer, which can contribute to optimizing applications in cooling technologies, microelectronics, and thermal management systems in energy applications [39,40,41]. In the early stages of research, Shit et al. [42] examined thermal transfer behavior of biofluids in microchannels with hydrophobic surfaces considering thermal radiation and Joule heating effects. Subsequently, Prakash et al. [43] investigated the dynamics of nanofluids driven by electroosmosis and peristalsis, taking into account the influence of thermal radiation. Mallick and Misra [44] studied the flow and heat transfer characteristics of nanofluids in hydrophobic cylindrical channels, considering nonlinear thermal radiation, particle size, and gravity. They found that nonlinear thermal radiation significantly influences the overall heat transfer in the channel. More recently, Saha and Kundu [45] delved into the impact of thermal radiation on the efficiency of electrokinetic energy conversion and thermal performance of couple-stress fluids characterized by SD zeta potential. Their findings demonstrated that thermal radiation parameters have a substantial impact on heat transfer. In all of these aforementioned studies, radiative heat transfer exerted a significant influence on the thermal efficiency of fluids in micro/nanochannels.

To enhance heat and mass transfer performance in micro/nanochannels, cylindrical micro/nanochannels have garnered significant attention [46,47,48]. Their high surface area-to-volume ratio provides higher heat transfer efficiency, particularly in microfluidic devices and high-efficiency heat exchangers [49]. Jian and his co-researchers [50,51,52] made significant contributions to the study of electroosmotic flow and heat transfer in cylindrical channels. They explored various fluid-driven mechanisms, including pressure, electroosmosis, external electric fields, and magnetic fields. They also analyzed the energy conversion performance and heat transfer efficiency of these cylindrical channels. Building on their research, Reshadi and Saidi [53] studied the effects of finite ion distribution and EDL overlap on the flow of nanofluids in cylindrical channels. Recently, considering interface slip and SD zeta potential, Deng et al. [54] studied the unstable two-layer pressure-driven electroosmotic flow in cylindrical microchannels. Nonetheless, the heat transfer efficiency of nanofluids in cylindrical channels under radiative heat transfer remains ambiguous when considering SD zeta potential.

To the best of our knowledge, previous studies have not explored the heat transfer properties of nanofluids within cylindrical hydrophobic microchannels subjected to thermal radiation. This study aims to examine the combined effects of EDL thickness, slip boundary conditions, and thermal radiation parameters on the heat transfer behavior of nanofluids in cylindrical hydrophobic microchannels. Initially, an approximate closed-form solution for the electric potential field is obtained by using the Debye–Huckel linearization assumption. This solution is then incorporated into the modified Navier–Stokes equation, and the analytical solution for the velocity field is determined, accounting for the coupled wall potential and slip conditions. Subsequently, using the energy conservation principle, analytical solutions for the temperature field are derived for both SD and SI zeta potentials. Finally, a comprehensive examination is conducted to evaluate the impact of various factors, including EDL thickness, nanoparticle volume fraction, slip length, thermal radiation parameters, and Brinkman number, on the Nusselt number.

## 2. Mathematical Modeling

**Figure 1:**
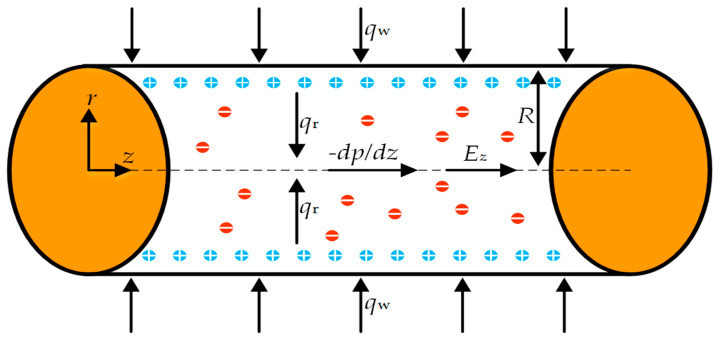
Schematic diagram of a cylindrical microchannel.

The nanofluid in a circular cylindrical microchannel with hydrophobic walls is depicted in Figure 1. Coordinates in a cylindrical framework are particularly useful for studying phenomena in circular microchannels. At the center of the microtube, a cylindrical coordinate system defined by (*r*, *φ*, *z*) is set up, with the *z*-axis aligned along the axial direction. The radius of the circular microchannel is *R*, and its length is *L*, where *L* ≫ 2*R*, indicating that the length is significantly greater than the diameter. The nanofluid flow is driven by an axial pressure gradient −*dp*/*dz*, and an applied external electric field *E_z_*. Additionally, a wall heat flux *q*_w_ is applied along the channel wall. The EDL forms near the liquid–wall interface. The mobile electrolyte ions are represented by small circles with + or − symbols. To simplify calculations while preserving the core physical principles, the following assumptions are made [45,55]:

- The wall heat flux *q*_w_ is consistent and unchanging along the channel wall;
- Compared to the radiative heat flux *q*_r_ in the *r* direction, the contribution of radiative heat transfer in the *z* direction is relatively small and does not significantly affect the overall thermal analysis. Therefore, it can be assumed to be negligible;
- The wall zeta potential is small enough for the Debye–Huckel linearization approximation to be applied;
- The flow is considered to be steady, laminar, thermally and hydrodynamically fully developed;
- Nanoparticles are uniformly dispersed within the base fluid and have reached thermal equilibrium with it. Consequently, the electric double layers formed around the nanoparticles are significantly smaller than those present on the channel walls;
- Because the designed Debye length of the pipe is significantly smaller than its diameter, it is assumed that the EDL formed on the channel walls do not overlap;
- Nanoparticles are assumed to be uniformly distributed.

### 2.1. Electric Potential

Using the Poisson–Boltzmann equation, the electric potential *ψ* of the EDL in the cylindrical coordinate system is given by [56]

(1) $$\frac{d^{2}\psi }{dr^{2}}+\frac{1}{r}\frac{d\psi }{dr}=-\frac{\rho_{e}}{\varepsilon }$$

where *ε* is the fluid permittivity, and *ρ*_e_ is the electric charge density. In the case of an ideal solution where a symmetric salt is fully dissociated, by using the Boltzmann distribution, the electric charge density is defined as [57]

(2) $$\rho_{e}=-2n_{0}eZ\sinh\left(\frac{eZ\psi }{k_{B}T_{\mathrm{av}}}\right)$$

where *n*_0_, *e*, *Z*, *k*_B_, and *T*_av_ denote the bulk ionic concentration, the elementary charge of an electron, the ionic valence present in the solution, the Boltzmann constant, and the average absolute temperature over the channel cross section, respectively.

Substituting Equation (2) into Equation (1) and using the Debye–Huckel approximation [58,59], the linear Poisson–Boltzmann equation is obtained as follows:

(3) $$\frac{d^{2}\psi }{dr^{2}}+\frac{1}{r}\frac{d\psi }{dr}=\frac{2n_{0}eZ}{\varepsilon }\left(\frac{eZ\psi }{k_{B}T_{\mathrm{av}}}\right)$$

According to the dimensionless parameters r^*^ = *r*/*R* and ψ^*^ = *eZψ*/*k*_B_*T*_av_, Equation (3) in the dimensionless form becomes

(4) $$\frac{d^{2}\psi^{*}}{dr^{*2}}+\frac{1}{r^{*}}\frac{d\psi^{*}}{dr^{*}}=k^{2}\psi^{*}$$

where *k* (=*κR*) is the electrokinetic width of the EDL, describing the ratio between the microchannel radius and the reciprocal of the EDL thickness and, *λ*_D_ = *κ*^−1^ = (*ԑk*_B_*T*_av_/2*n*_0_*e*^2^*Z*^2^)^1/2^ is the Debye length.

When considering the hydrophobic channels as wall surfaces, the boundary conditions corresponding to the potential field at the wall are also altered, requiring the consideration of the slip dependence on the zeta potential. In the early stages of studying this coupled phenomenon, Yang and Kwok [60] proposed a simple linear relationship between the zeta potential and slip conditions under the parallel plate model. Subsequent studies extended this linear result to cylindrical and rectangular pipe geometries [61]. Based on the previous literature [16,18,20], the relationship between the apparent zeta potential $\zeta_{\beta }$ and the slip length *β* can be expressed as follows:

(5) $$\zeta_{\beta }=\zeta \left\{1+\beta \kappa \frac{\sinh\left(\zeta eZ/k_{B}T_{\mathrm{av}}\right)}{\zeta eZ/k_{B}T_{\mathrm{av}}}\right\}$$

By introducing the following dimensionless conditions, $\zeta^{*}=\zeta eZ/k_{B}T_{\mathrm{av}}$, $\zeta_{\beta }^{*}=\zeta_{\beta }eZ/k_{B}T_{\mathrm{av}}$, and $\beta^{*}=\beta /R$, Equation (5) can be transformed into the following dimensionless form:

(6) $$\zeta_{\beta }^{*}=\zeta^{*}\left\{1+\beta^{*}k\frac{\sinh\left(\zeta^{*}\right)}{\zeta^{*}}\right\}$$

where β* is the dimensionless slip length, ζ* is the zeta potential for the no-slip case, and ζ* = 1 under the Debye–Huckel linear approximation [62].

The boundary conditions for Equation (4) are obtained as

(7) $${\left(\psi^{*}\right)}_{r^{*}=1}=\zeta_{\beta }^{*},\;{\left(\frac{d\psi^{*}}{dr^{*}}\right)}_{r^{*}=0}=0$$

Using the governing equation, Equation (4), and boundary conditions Equation (7), the analytical solution of the SD EDL potential can be obtained as

(8) $$\psi^{*}\left(r^{*}\right)=\zeta_{\beta }^{*}\frac{I_{0}\left(kr^{*}\right)}{I_{0}\left(k\right)}$$

where *I*_0_ is the zeroth-order modified Bessel function of the first kind.

### 2.2. Velocity Field

In conducting this analysis axially, symmetric flow is considered, with the velocity field dependent on the radial coordinate *r*. Fluid velocity, as dictated by the modified Navier–Stokes equations, is expressed as [10]

(9) $$-\frac{dp}{dz}+\mu_{\mathrm{eff}}\left(\frac{d^{2}u}{dr^{2}}+\frac{1}{r}\frac{du}{dr}\right)+\rho_{e}E_{z}=0$$

where *u* is the axial velocity, *p* is the axial pressure, *ρ*_e_*E_z_* represents the body force induced by electroosmotic actuation, and *μ*_eff_ is the effective viscosity of the nanofluid, which is defined as [63]

(10) $$\mu_{\mathrm{eff}}=\frac{\mu_{f}}{{\left(1-\phi \right)}^{2.5}}$$

where *μ*_f_ is the viscosity of the base fluids, and *ϕ* is the volume fraction of nanoparticles.

Using Equation (1) in Equation (9),we can obtain

(11) $$-\frac{1}{\mu_{\mathrm{eff}}}\frac{dp}{dz}+\left(\frac{d^{2}u}{dr^{2}}+\frac{1}{r}\frac{du}{dr}\right)-\frac{\varepsilon E_{z}}{\mu_{\mathrm{eff}}}\left(\frac{d^{2}\psi }{dr^{2}}+\frac{1}{r}\frac{d\psi }{dr}\right)=0$$

Introduce the following dimensionless parameters:

(12) $$u^{*}=\frac{u}{u_{\mathrm{HS}}},\;G=-\frac{dp}{dz}\frac{R^{2}}{\mu_{f}u_{\mathrm{HS}}},\;u_{\mathrm{HS}}=-\frac{\varepsilon k_{B}T_{\mathrm{av}}E_{z}}{\mu_{f}eZ},\;\eta =\frac{\mu_{f}}{\mu_{\mathrm{eff}}}$$

where *u*_HS_ represents the Helmholtz–Smoluchowski electroosmotic velocity, *G* is the velocity scale representing the ratio of the maximum velocity of pressure-driven flow in a channel to the Helmholtz–Smoluchowski electroosmotic velocity.

Using the above dimensionless parameters, the governing equation, Equation (11), and the corresponding boundary conditions can be obtained as

(13) $$\frac{d^{2}u^{*}}{dr^{{*}^{2}}}+\frac{1}{r^{*}}\frac{du^{*}}{dr^{*}}+\eta G+\eta k^{2}\psi^{*}=0$$

and

(14) $${\left(u^{*}\right)}_{r^{*}=1}=-\beta^{*}{\left(\frac{du^{*}}{dr^{*}}\right)}_{r^{*}=1},\;{\left(\frac{du^{*}}{dr^{*}}\right)}_{r^{*}=0}=0$$

By solving the governing equation, Equation (13), and boundary conditions Equation (14), the analytical solution for the fluid velocity with SD zeta potential can be obtained as follows:

(15) $$u^{*}(r^{*})=A_{1}+A_{2}\frac{I_{0}(kr^{*})}{I_{0}(k)}+A_{3}r^{*2}$$

where $A_{1}=-A_{3}\left(1+\beta^{*}\right)-A_{2}\left[1+\frac{\beta^{*}kI_{1}(k)}{I_{0}(k)}\right]$, $A_{2}=-\eta \zeta_{\beta }^{*}$, $A_{3}=-\frac{1}{4}\eta G$, and *I*_1_() is the first-order modified Bessel function of the first kind.

If the effect of the SD zeta potential on the fluid velocity is not considered, i.e., $\zeta_{\beta }^{*}=\zeta^{*}$, Equation (15) reduces to the expression for the fluid velocity corresponding to the SI zeta potential.

### 2.3. Temperature Field

According to the velocity distribution of electroosmotic flow, the thermal transport properties of a circular microchannel under radiative heat flux *q*_r_ can be further investigated. Viscous dissipation, caused by fluid flow resistance, has a substantial impact on the overall thermal behavior, and the addition of radiative heat flux complicates the heat transfer process, especially at high temperatures. Therefore, these factors must be considered in the energy equation. By accounting for axial conduction, volumetric Joule heating, viscous dissipation, and thermal radiation, the energy conservation equation in the cylindrical coordinate system can be expressed as [64]

(16) $${\left(\rho C_{p}\right)}_{\mathrm{eff}}u\frac{\partial T}{\partial z}=k_{\mathrm{eff}}\left[\frac{1}{r}\frac{\partial }{\partial r}\left(r\frac{\partial T}{\partial r}\right)+\frac{\partial^{2}T}{\partial z^{2}}\right]+\sigma_{e}E_{z}^{2}+\mu_{\mathrm{eff}}{\left(\frac{du}{dr}\right)}^{2}-\frac{1}{r}\frac{\partial }{\partial r}\left(rq_{r}\right)$$

where *T* is the temperature of the fluid, *σ*_e_ is the electrical conductivity of the nanofluid, and (*ρC*_p_)_eff_ is the effective heat capacity of nanofluids under a reference pressure, which can be written as [65]

(17) $${\left(\rho C_{p}\right)}_{\mathrm{eff}}={\left(\rho C_{p}\right)}_{f}(1-\phi )+{\left(\rho C_{p}\right)}_{s}\phi$$

where (*ρC*_p_)_s_ and (*ρC*_p_)_f_, respectively, represent the heat capacitance of the nanoparticles and the base fluids.

The effective thermal conductivity of nanofluids *k*_eff_ is given as [27]

(18) $$\;k_{\mathrm{eff}}=k_{f}\left(\frac{k_{s}+2k_{f}+2\left(k_{s}-k_{f}\right){\left(1+\omega \right)}^{3}\phi }{k_{s}+2k_{f}-\left(k_{s}-k_{f}\right){\left(1+\omega \right)}^{3}\phi }\right)$$

where *k*_s_ and *k*_f_, respectively, represent the thermal conductivity of the nanoparticles and the base fluid, and *ω* is a dimensionless parameter expressed as the ratio of the nanoparticle layer thickness to the initial particle radius. Typically, the value of *ω* is assumed to be 0.1 [66]. On the right-hand side of Equation (16), the first term represents axial thermal conduction, the second term represents the volumetric heat generation due to Joule heating effects, the third term represents the viscous dissipation, and the last term represents the thermal radiation effects.

According to the Rosseland approximation, the radiation heat flux is given as [67]

(19) $$q_{r}=-\frac{4\sigma }{3k^{*}}\frac{\partial T^{4}}{\partial r}$$

where *σ* is the Stefan–Boltzmann constant, and k* is the average absorption coefficient. The term *T*^4^ can be expanded to about the wall temperature *T*_w_ in a power series. By neglecting the higher order terms, *T*^4^ can be obtained as

(20) $$T^{4}\approx 4T_{w}^{3}T-3T_{w}^{4}$$

Applying the characteristics of thermally fully developed flow with the condition of constant heat flux on the wall leads to

(21) $$\frac{\partial T}{\partial z}=\frac{dT_{w}}{dz}=\frac{dT_{m}}{dz}=\mathrm{const}.\;\mathrm{and}\;\frac{\partial^{2}T}{\partial z^{2}}=0$$

where *T*_m_ is the bulk mean temperature.

By applying the energy balance equation to a volume element of length *dz* and combining it with Equations (16) and (21), we obtain

(22) $$\frac{\partial T}{\partial z}=\frac{1}{{\left(\rho C_{p}\right)}_{\mathrm{eff}}Ru_{m}}\left[2q_{w}+R\sigma_{e}E_{z}^{2}+\frac{2\mu_{\mathrm{eff}}{u_{\mathrm{HS}}}^{2}}{R}\int_{0}^{1}{\left(\frac{du^{*}}{dr^{*}}\right)}^{2}r^{*}dr^{*}+\frac{\sigma 16{T_{w}}^{3}}{3k^{*}k_{\mathrm{eff}}}2q_{w}\right]$$

where $q_{w}=k_{\mathrm{eff}}{\left(\frac{\partial T}{\partial r}\right)}_{r=R}$ is the wall heat flux, and *u*_m_ is the mean axial velocity, which can be determined by integrating *u*(*r*) across the cross-sectional area of the microchannel

(23) $$u_{m}=\frac{1}{\pi R^{2}}\int_{0}^{2\pi }\int_{0}^{R}u\left(r\right)rdrd\varphi =2u_{\mathrm{HS}}\beta_{1}$$

in which $\beta_{1}=\int_{0}^{1}u^{*}r^{*}dr^{*}$.

Introduce the following dimensionless parameters:

(24) $$\theta (r^{*})=\frac{k_{f}(T-T_{w})}{q_{w}R},\;\beta_{2}=\int_{0}^{1}{\left(\frac{du^{*}}{dr^{*}}\right)}^{2}r^{*}dr^{*},\;S=\frac{\sigma_{e}E_{z}^{2}R}{q_{w}},\;Br=\frac{\mu_{f}u_{\mathrm{HS}}^{2}}{q_{w}R},\;Nr=\frac{16\sigma T_{w}^{3}}{3k^{*}k_{f}}$$

where *S* is the Joule heating parameter, *Br* is the Brinkman number, and *Nr* is the radiative heating parameter.

According to Equations (16) and (22)–(24), the dimensionless energy conservation equation and the corresponding boundary conditions can be given as

(25) $$\left(1+Nr\frac{k_{f}}{k_{\mathrm{eff}}}\right)\left(\frac{d^{2}\theta }{dr^{*2}}+\frac{1}{r^{*}}\frac{d\theta }{dr^{*}}\right)=\frac{k_{f}}{k_{\mathrm{eff}}}\left(u^{*}\frac{1}{\beta_{1}}\left[\left(1+Nr\lambda \right)+\frac{S}{2}+\frac{Br}{\eta }\beta_{2}\right]-S-\frac{Br}{\eta }{\left(\frac{du^{*}}{dr^{*}}\right)}^{2}\right)$$

(26) $${\left(\frac{d\theta }{dr^{*}}\right)}_{r^{*}=0}=0,\;{\left(\frac{d\theta }{dr^{*}}\right)}_{r^{*}=1}=\frac{k_{f}}{k_{\mathrm{eff}}}\left(\mathrm{or}{\left(\theta \right)}_{r^{*}=1}=0\right)$$

By solving Equations (25) and (26), the analytical solution for the dimensionless temperature field with SD zeta potential can be obtained as follows:

(27) $$\begin{array}{l} \theta \left(r^{*}\right)=\frac{1}{16}\left(r^{*2}-1\right)\left[4\left(A_{1}B_{2}+B_{1}\right)+A_{3}\left(4A_{3}B_{3}+B_{2}\right)\left(r^{*2}+1\right)\right]\\ +\frac{A_{2}}{2k^{2}I_{0}(k)}\left\{B_{3}k\left[8A_{3}rI_{1}(kr^{*})-\left(8A_{3}+A_{2}k^{2}\right)I_{1}(k)\right]+2(B_{2}-8A_{3}B_{3})I_{0}(kr^{*})\right\}\\ +\frac{A_{2}^{2}B_{3}}{2I_{0}{\left(k\right)}^{2}}\left\{k^{2}\left[r^{2}I_{1}{\left(kr^{*}\right)}^{2}-I_{1}{\left(k\right)}^{2}\right]+\left(1-k^{2}r^{*2}\right)I_{0}{\left(kr^{*}\right)}^{2}+krI_{0}(kr^{*})I_{1}(kr^{*})\right\}\\ +\frac{A_{2}(8A_{3}B_{3}-B_{2})}{k^{2}}+\frac{1}{2}A_{2}^{2}B_{3}\left(k^{2}-1\right) \end{array}$$

where $\lambda =\frac{k_{f}}{k_{\mathrm{eff}}}$, $B_{1}=B_{4}M$, $B_{2}=-B_{4}S$, $B_{3}=-\frac{B_{4}Br}{\eta }$, $B_{4}=\frac{\lambda }{\lambda Nr+1}$, $M=\frac{1}{\beta_{1}}\left(1+\lambda Nr+\frac{S}{2}+\frac{\beta_{2}Br}{\eta }\right)$.

If the effect of the SD potential on the temperature field is neglected, i.e., $\zeta_{\beta }^{*}=\zeta^{*}$, Equation (27) reduces to the temperature field for the SI zeta potential.

The Nusselt number, which characterizes the intensity of heat transfer, is defined as [68]

(28) $$Nu=\frac{2q_{w}R}{k_{\mathrm{eff}}(T_{w}-T_{m})}$$

The dimensionless overall temperature is defined as follows [69]:

(29) $$\theta_{m}=\frac{\int_{0}^{2\pi }\int_{0}^{1}u^{*}\theta r^{*}dr^{*}d\varphi }{\int_{0}^{2\pi }\int_{0}^{1}u^{*}r^{*}dr^{*}d\varphi }=\frac{k_{f}\left(T_{m}-T_{w}\right)}{q_{w}R}$$

Substituting Equation (28) into Equation (29), the Nusselt number can be given as follows:

(30) $$Nu=-\frac{k_{f}}{k_{\mathrm{eff}}}\frac{2}{\theta_{m}}$$

Using the velocity from Equation (15) and temperature distributions from Equation (27), the Nusselt number can be calculated using Equation (30).

## 3. Results and Discussions

In our computations, the relevant parameters are presented in Table 1. Referring to the relevant comprehensive literature [28], the thickness of the EDL in a symmetric Al_2_O_3_ electrolyte solution is approximately 300 nm. Under the assumption of non-overlapping EDL, the radius of the microtube needs to be set in the range of 3–30 μm. Therefore, the range of the dimensionless parameter *k* is determined to be 10–100. Additionally, the ranges of other parameters are given as follows: dimensionless volume fraction of nanoparticles *ϕ*~0–0.08 [70], dimensionless slip length β^*^~0–0.01 [71], Brinkman number *Br*~0.001–0.1 [19], dimensionless Joule heating parameter *S*~0–5 [72], and dimensionless thermal radiation parameter *Nr*~0–5 [45].

### 3.1. Validation of Results

**Figure 2:**
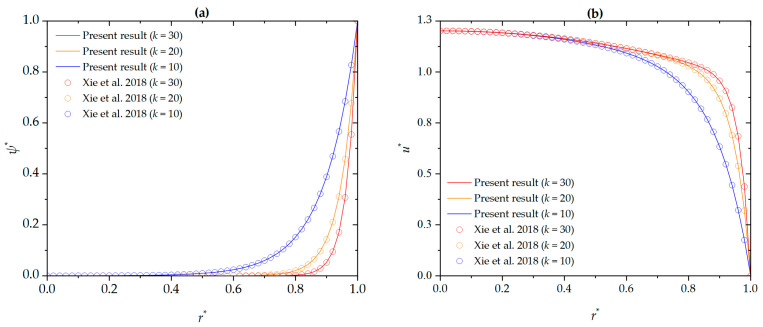
Comparison of the dimensionless (**a**) electric potential and (**b**) velocity distribution [52].

In the present study, analytical solutions have been derived to evaluate the electric potential, flow velocity, and temperature fields of the nanofluid (Al_2_O_3_-water) through cylindrical microchannels under the effects of pressure-driven flow, electroosmotic forces, and fully developed thermal conditions. To verify the accuracy of the proposed model, we compare the dimensionless electric potential and velocity distributions with those of Xie et al. [52] for different EDL electrokinetic widths (the inverse of the EDL thickness), as shown in Figure 2. In Figure 2a, the electric potential distribution for the SI zeta potential (*k* = 10, 30, 50, β^*^ = 0) is presented. The comparison results show excellent agreement. Furthermore, the comparison of the velocity distribution without considering slip and nanoparticle volume fraction conditions (*k* = 10, 30, 50, β^*^ = 0, *G* = 1, *ϕ* = 0) is shown in Figure 2b. The comparison results from this figure also match well. Therefore, our model accurately reproduces the findings reported in the literature.

### 3.2. Velocity Distribution

**Figure 3:**
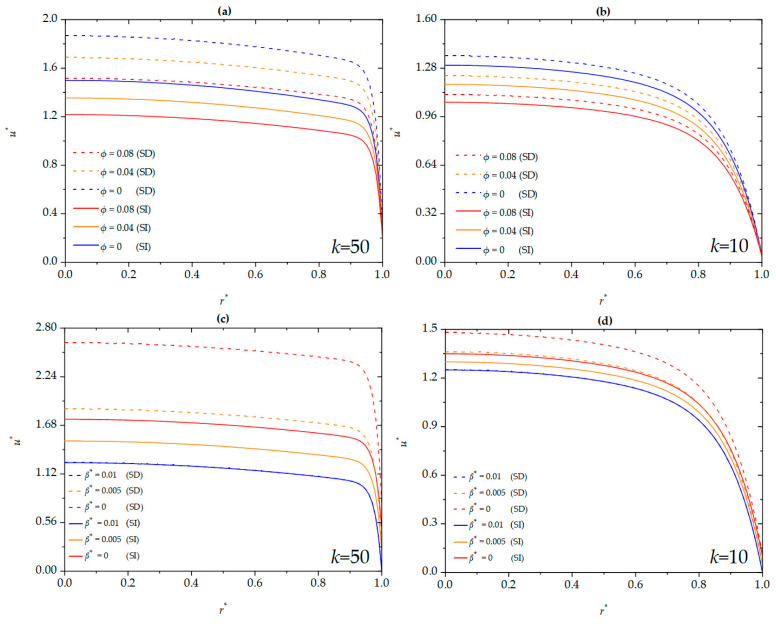
The dimensionless velocity for different values of *ϕ* and β* at (**a**) *k* = 50, β^*^ = 0.005; (**b**) *k* = 10, β* = 0.005; (**c**) *k* = 50, *ϕ* = 0; (**d**) *k* = 10, *ϕ* = 0 when *G* = 1.

The current section explores the influence of parameters such as EDL thickness, nanoparticle volume fraction, and dimensionless slip length on the dimensionless velocity in a cylindrical microchannel for both SD and SI zeta potentials, as shown in Figure 3. It is found that, for both SD and SI cases, the dimensionless velocity of the nanofluid decreases with an increase in nanoparticle volume fraction and increases with an enhanced dimensionless slip length for different EDL thicknesses (thinner with *k* = 50 and thicker with *k* = 10) and decreases with an increase in nanoparticle volume fraction and increases with an enhanced dimensionless slip length. Notably, compared to the SI zeta potential, the dimensionless velocity is higher for the SD zeta potential, which is consistent with findings in planar microchannels [19]. This enhancement is attributed to the increased hydrophobicity near the walls under slip conditions, which reduces the frictional resistance of the flow, allowing the fluid to move at higher speeds. Additionally, under the condition of a thinner EDL (*k* = 50), the slip length exerts a more significant impact on the dimensionless velocity of the nanofluid, especially in close proximity to the walls.

### 3.3. Temperature Distribution

**Figure 4:**
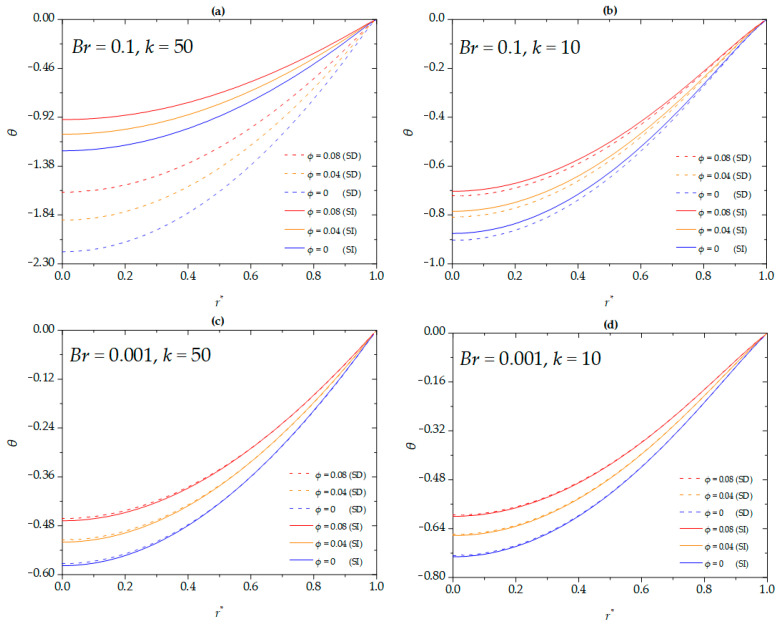
The dimensionless temperature for different values of *ϕ* at (**a**) *Br* = 0.1, *k* = 50; (**b**) *Br* = 0.1, *k* = 10; (**c**) *Br* = 0.001, *k* = 50; (**d**) *Br* = 0.001, *k* = 10 when β^*^ = 0.01, *G =* 1, *S =* 5, *Nr* = 0.

The effects of nanoparticle volume fraction on the dimensionless temperature of the nanofluid for different Brinkman numbers and EDL thicknesses under SD and SI zeta potentials are shown in Figure 4. The magnitude of the dimensionless temperature decreases with increasing nanoparticle volume fraction for both SI and SD conditions. For a higher Brinkman number (*Br* = 0.1), the variation in dimensionless temperature between the SD and SI cases is significant. Specifically, the magnitude of the dimensionless temperature for the SD condition is higher than that for the SI condition. This is because, at higher Brinkman numbers, higher flow speeds result in greater viscous dissipation. However, SD conditions significantly enhance the flow speed of the nanofluid, creating a larger velocity gradient, which leads to greater viscous heating and higher temperatures compared to the SI condition. In contrast, this trend is reversed at a lower Brinkman number (*Br* = 0.001). The SD zeta potential increases the flow rate of the nanofluid, thereby enhancing the convective heat transfer capabilities of the microchannel. The effects of viscous dissipation are minimal. Additionally, for a thinner EDL (*k* = 50), the difference between SD and SI zeta potential conditions becomes more pronounced compared to a thicker EDL (*k* = 10).

**Figure 5:**
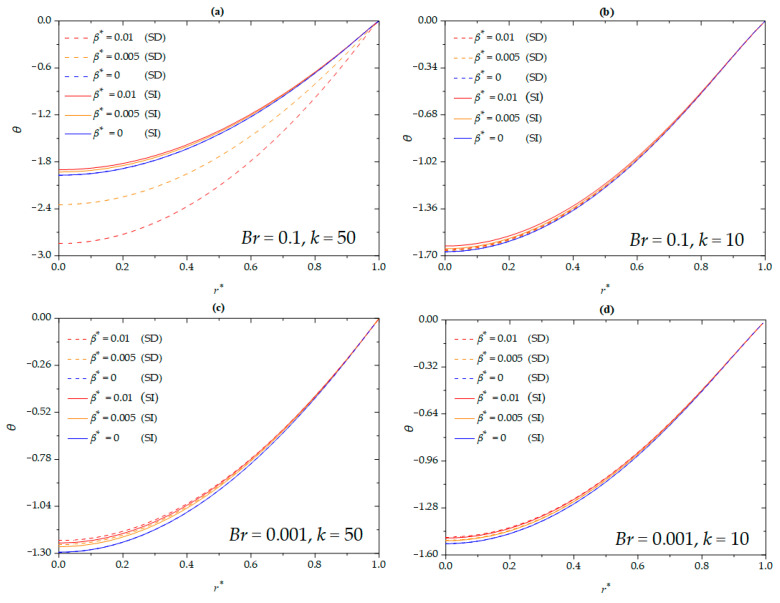
The dimensionless temperature for different values of β^*^ at (**a**) *Br* = 0.1, *k* = 50; (**b**) *Br* = 0.1, *k* = 10; (**c**) *Br* = 0.001, *k* = 50; (**d**) *Br* = 0.001, *k* = 10 when *ϕ* = 0, *G* = 1, *S* = 5, *Nr* =1.

Figure 5 shows the influence of the dimensionless slip length on the dimensionless temperature of the nanofluid for different Brinkman numbers and EDL thicknesses under both SD and SI zeta potentials. Under the conditions of a higher Brinkman number (*Br* = 0.1) and thinner EDL thickness (*k* = 50), the dimensionless slip length exerts a considerable influence on the magnitude of the dimensionless temperature for the SD case compared to the SI case. This result indicates that, for a higher Brinkman number or thinner EDL thickness, the acceleration of velocity leads to greater viscous dissipation owing to the enhancement of the apparent zeta potential. Furthermore, under the condition of thinner EDL (*k* = 50), the magnitude of the dimensionless temperature increases with increasing dimensionless slip length for the SD zeta potential, while a contrary trend is observed under the condition of thicker EDL (*k* = 10). This result is consistent with findings in planar microchannels [19]. Notably, this trend is also observed at the lower Brinkman number (*Br* = 0.001), which is similar to the results discussed above (see Figure 4).

**Figure 6:**
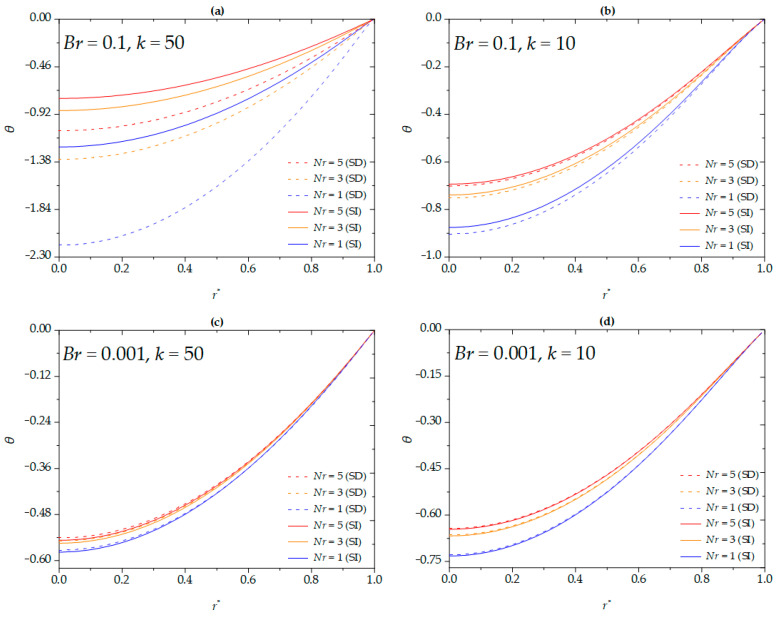
The dimensionless temperature for different values of *Nr* at (**a**) *Br* = 0.1, *k* = 50; (**b**) *Br* = 0.1, *k* = 10; (**c**) *Br* = 0.001, *k* = 50; (**d**) *Br* = 0.001, *k* = 10 when β^*^ = 0.01, *G =* 1, *S =* 5, *ϕ* = 0.

Figure 6 illustrates the impact of the radiative heat parameter on the dimensionless temperature of the nanofluid for different Brinkman numbers and EDL thicknesses under SD and SI zeta potentials. The magnitude of the dimensionless temperature decreases with an increase in the radiative heat parameter, regardless of whether the conditions are SI or SD zeta potentials. At higher Brinkman number (*Br* = 0.1), the variation between SD and SI zeta potentials is significant, especially at thinner EDL thickness (*k* = 50), which is consistent with the trends observed in Figure 4a and Figure 5a. Moreover, the magnitude of the dimensionless temperature for the SD zeta potential is higher than that for the SI zeta potential, which is due to the greater viscous dissipation caused by the higher flow velocity associated with the SD zeta potential. Similar to the results in Figure 4c,d, as well as Figure 5c,d, at a lower Brinkman number, the magnitude of the dimensionless temperature for the SD zeta potential is lower than that for the SI zeta potential. This implies that when considering the use of hydrophobic materials for the wall surfaces, the magnitude of the dimensionless temperature of the nanofluid is often overestimated.

### 3.4. Nusselt Number

**Figure 7:**
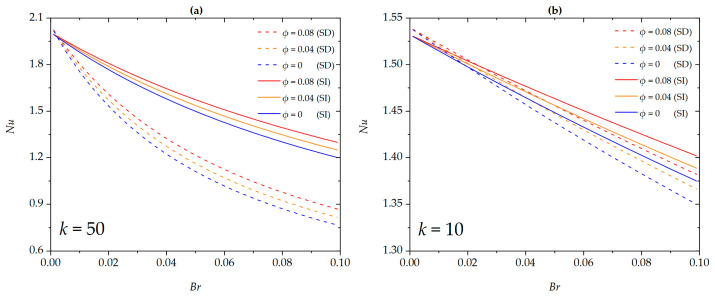
The Nusselt number for different values of *ϕ* at (**a**) *k* = 50; (**b**) *k* = 10 when *G* = 1, *S =* 5, β* = 0.01, *Nr* = 1.

Figure 7 investigates the variation trends of the Nusselt number with regard to Brinkman number and nanoparticle volume fraction under SD and SI zeta potential conditions for different EDL thicknesses. The Nusselt number shows a decreasing trend as the Brinkman number increases. This is because as the Brinkman number increases, the effect of viscous dissipation on the nanofluid intensifies, raising the wall temperature. Consequently, the average dimensionless temperature of the nanofluid decreases, leading to a reduction in the Nusselt number as the Brinkman number increases. Additionally, as the nanoparticle volume fraction increases, the Nusselt number increases. The presence of nanoparticles within the nanofluid enhances the fluid’s heat transfer capability, thereby strengthening the convective heat transfer intensity of the microchannel. The nanoparticle volume fraction reveals a significant positive impact on improving the Nusselt number. Furthermore, at lower Brinkman numbers, there exists an intersection point between the Nusselt numbers corresponding to SD and SI zeta potentials for different nanoparticle volume fractions. Before the intersection point, the Nusselt number for the SD zeta potential is generally lower than that for the SI zeta potential, while after the intersection point, the trend reverses. This observation aligns with the phenomena shown in Figure 4.

**Figure 8:**
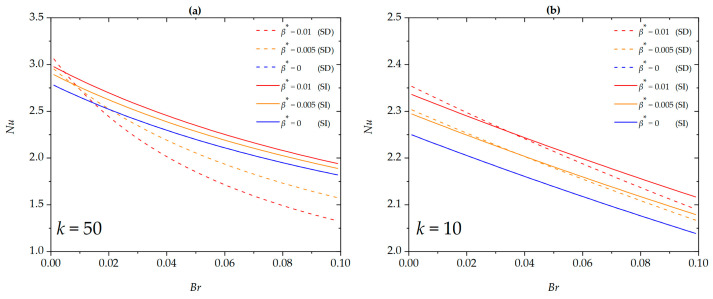
The Nusselt number for different values of β* at (**a**) *k* = 50; (**b**) *k* = 10 when *G* = 1, *S =* 5, *ϕ* = 0, *Nr* = 1.

The correlation between the Nusselt number with the Brinkman number and the dimensionless slip length under SD and SI zeta potential conditions for different EDL thicknesses is presented in Figure 8. This enhancement is observed under the conditions of a thinner EDL (*k* = 50) and SD zeta potential, where the Nusselt number decreases with increasing dimensionless slip length; conversely, an opposite trend is observed for a thicker EDL (*k* = 10). This is because, under the SD zeta potential and thinner EDL, the viscous heating effect is amplified near the wall, raising the wall temperature, which in turn reduces the Nusselt number. Moreover, similar to the results discussed above (see Figure 7), there exists an intersection point between the Nusselt numbers corresponding to SD and SI zeta potentials for different dimensionless slip lengths. This observation aligns with the phenomena shown in Figure 5.

**Figure 9:**
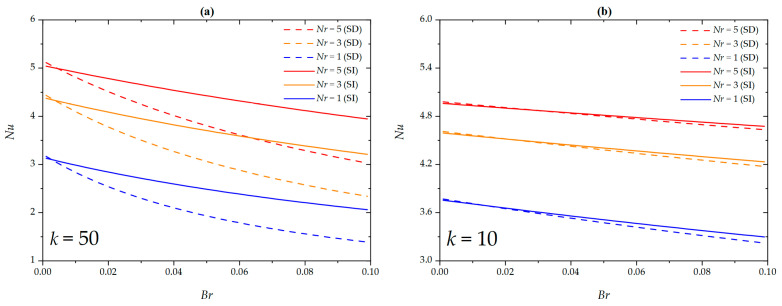
The Nusselt number for different values of *Nr* at (**a**) *k* = 50; (**b**) *k* = 10 when *G* = 1, *S =* 5, *ϕ* = 0, β* = 0.01.

Figure 9 examines the variation of the Nusselt number with the radiative heat parameter under SD and SI zeta potential conditions for different EDL thicknesses. It is observed that as the radiative heat parameter increases, the Nusselt number also increases. This is because the radiative heat reduces the temperature variation between the wall and the fluid, thereby lowering the dimensionless average temperature and enhancing the convective heat transfer capability within the microchannel. Additionally, an intersection point exists between the Nusselt numbers corresponding to SD and SI zeta potentials at lower Brinkman numbers, which corresponds to the results shown in Figure 6. Furthermore, under the condition of a thinner EDL (*k* = 50), the difference in Nusselt numbers between SD and SI conditions becomes more pronounced.

## 4. Conclusions

At present, we analyze the flow and heat transfer characteristics of a nanofluid (Al_2_O_3_-water) in cylindrical microchannels, considering the effects of thermal radiation and SD zeta potential under external electric field and pressure-driven flow conditions. By solving the linearized Poisson–Boltzmann equation, the modified Navier–Stokes equation, and the energy conservation equation, we analytically investigate the electric potential field, velocity field, temperature field, and Nusselt number. The influences of various factors, including EDL thickness, nanoparticle volume fraction, dimensionless slip length, thermal radiation parameter, and Brinkman number, are discussed. The main conclusions are as follows:

1. As the nanoparticle volume fraction increases, the flow velocity decreases, while both the dimensionless temperature and the Nusselt number increase for both SD and SI zeta potentials, thereby enhancing the fluid’s heat transfer capability and significantly improving heat transfer performance;
2. The flow velocity increases as the dimensionless slip length increases. Except for the case of thinner EDL and SD zeta conditions, the dimensionless temperature and Nusselt number increase with the dimensionless slip length for both SD and SI cases;
3. As the thermal radiation parameter increases, the dimensionless temperature of the fluid rises, with higher temperatures observed under the SD condition. Simultaneously, the Nusselt number increases with the thermal radiation parameter but decreases with an increase in the Brinkman number;
4. An intersection point exists between the Nusselt numbers corresponding to SD and SI zeta potentials at lower Brinkman numbers;
5. Under the condition of thinner EDL, the differences in flow velocity, temperature, and Nusselt number between SD and SI conditions become more pronounced.

## Figures and Tables

**Table 1:** Physical properties in microchannels [70,73,74].

Parameters	Symbol	Values	Units
Thermal conductivity of the nanoparticles	*k*_s_	40	W·m^−1^·K^−1^
Thermal conductivity of the base fluid	*k*_f_	0.63	W·m^−1^·K^−1^
Viscosity of the base fluids	*μ*_f_	8.91 × 10^−3^	kg·m^−1^·s^−1^
Boltzmann constant	*k*_B_	1.381 × 10^−23^	J·K^−1^
Average absolute temperature	*T*_av_	298~300	K
Radius of the microchannel	*R*	3~100	μm
Fluid’s electric permittivity	*ԑ*	6.95 × 10^−10^	C·V^−1^·m^−1^
Pressure gradient	−*dp*/*dz*	10~100	kPa·m^−1^
Electric field	*E_z_*	0~10	kV·m^−1^
Electroosmotic velocity	*u*_HS_	100	μm·s^−1^
Stefan–Boltzmann constant	*σ*	5.67 × 10^−8^	W·m^−2^·K^−4^
Average absorption coefficient	*k* ^*^	0.1~100	m^−1^
Wall temperature	*T*_w_	298~300	K

## Data Availability

The data presented in this study are available on request from the corresponding author.
